# The Clinical Significance of HbA1c in Operable Chronic Thromboembolic Pulmonary Hypertension

**DOI:** 10.1371/journal.pone.0152580

**Published:** 2016-03-31

**Authors:** Manuel Jonas Richter, Katrin Milger, Sarah Haase, Natascha Sommer, Khodr Tello, Werner Seeger, Eckhard Mayer, Christoph Benjamin Wiedenroth, Friedrich Grimminger, Wolfgang George, Hossein Ardeschir Ghofrani, Stefan Guth, Henning Gall

**Affiliations:** 1 Department of Pneumology, Kerckhoff Heart, Rheuma and Thoracic Center, Bad Nauheim, Germany; 2 Department of Internal Medicine, Justus-Liebig-University Giessen, Universities of Giessen and Marburg Lung Center (UGMLC), Member of the German Center for Lung Research (DZL), Giessen, Germany; 3 Medical Clinic V, University of Munich, Comprehensive Pneumology Center, Member of the German Center for Lung Research, Munich, Germany; 4 Department of Thoracic Surgery, Kerckhoff Heart, Rheuma and Thoracic Center, Bad Nauheim, Germany; 5 TransMIT Project Division for Health Services Research and Consulting, THM University of Applied Science, Giessen, Germany; Indiana University, UNITED STATES

## Abstract

**Background:**

Glycosylated hemoglobin A1c (HbA1c) has been proposed as an independent predictor of long-term prognosis in pulmonary arterial hypertension. However, the clinical relevance of HbA1c in patients with operable chronic thromboembolic pulmonary hypertension (CTEPH) remains unknown. The aim of the present study was to investigate the clinical significance of HbA1c as a biomarker in CTEPH.

**Methods:**

Prospectively, 102 patients underwent pulmonary endarterectomy (PEA) in our national referral center between March 2013 and March 2014, of which after exclusion 45 patients were analyzed. HbA1c- levels, hemodynamic and exercise parameters were analyzed prior and one-year post-PEA.

**Results:**

45 patients (BMI: 27.3 ± 6.0 kg/m^2^; age: 62.7 ± 12.3 years) with a mean pulmonary arterial pressure (mPAP) of 43.6 ± 9.4 mmHg, a pulmonary vascular resistance (PVR) of 712.1 ± 520.4 dyn*s/cm^5^, a cardiac index (CI) of 2.4 ± 0.5 l/min/m^2^ and a mean HbA1c-level of 39.8 ± 5.6 mmol/mol were included. One-year post-PEA pulmonary hemodynamic and functional status significantly improved in our cohort. Baseline HbA1c-levels were significantly associated with CI, right atrial pressure, peak oxygen uptake and the change of 6-minute walking distance using linear regression analysis. However, using logistic regression analysis baseline HbA1c-levels were not significantly associated with residual post-PEA PH.

**Conclusions:**

This is the first prospective study to describe an association of HbA1c-levels with pulmonary hemodynamics and exercise capacity in operable CTEPH patients. Our preliminary results indicate that in these patients impaired glucose metabolism as assessed by HbA1c is of clinical significance. However, HbA1c failed as a predictor of the hemodynamic outcome one-year post-PEA.

## Introduction

Chronic thromboembolic pulmonary hypertension (CTEPH) is caused by unresolved pulmonary vascular obstruction due to recurrent embolism, resulting in an increase in mean pulmonary arterial pressure (mPAP) and pulmonary vascular resistance (PVR) [1, 2]. The progression of the disease is thought to be mainly caused by secondary small-vessel arteriopathy in the non-obstructed areas and concomitant right ventricle (RV) dysfunction [1, 2]. Untreated, the disease may progress towards progressive right ventricle loading, hypertrophy and failure [1, 2]. In case of surgically accessible CTEPH, pulmonary endarterectomy (PEA) offers a potentially curative treatment with a high survival rate and an excellent long-term outcome [3, 4]. However, residual post-PEA pulmonary hypertension (PH) has been recognized as main determinant for mortality [5] and impaired exercise capacity [6]. Predictors of functional outcome post-PEA are important in daily clinical practice. Therefore, identifying clinically relevant biomarkers, which are indicative of the functional outcome after PEA, are of high interest in operable CTEPH. Predictors of a favorable outcome post-PEA include pre-operative forced expiratory volume in 1s (FEV1), heart-type fatty acid-binding protein (H-FABP) and cardiac index (CI) [7, 8]. In most cases the residual PH results from a combination of concomitant small vessel disease, incomplete removal of obstructions and varying degree of reverse right ventricular remodeling after surgery [9]. Thus, the precise characterization of the contribution of large and small vessel disease in CTEPH is mandatory for the indication and outcome after PEA [10]. Recently, metabolic disorders have been identified as negative prognostic factors in pulmonary arterial hypertension (PAH) [11,12]. Interestingly, glycosylated hemoglobin A1c (HbA1c) has been reported as a significant biomarker in PAH [11, 13] and insulin resistance (IR) appears to be a risk factor and/or disease modifier that might impact survival in PAH [14]. In the general population, increased HbA1c-levels are strongly associated with microvascular complications [15]. The glycemic environment has been found to cause vascular damage due to chronic inflammation, oxidative stress and endothelial dysfunction [16]. In addition, non-diabetic hyperglycemia is an independent risk factor for cardiovascular disorders [17]. Pathophysiological IR and dysregulated glucose metabolism may be modifiers of disease in PH due to enhancement of inflammatory processes, dysregulation of the nitric oxide (NO) pathway and endothelial damage [13, 18]. Therefore, we hypothesized that the concomitant secondary small-vessel arteriopathy in CTEPH might be influenced by the glycemic environment and HbA1c-levels prior to PEA and might help to identify patients with a favorable functional outcome post-PEA.

## Methods

### Patients

**Fig 1:**
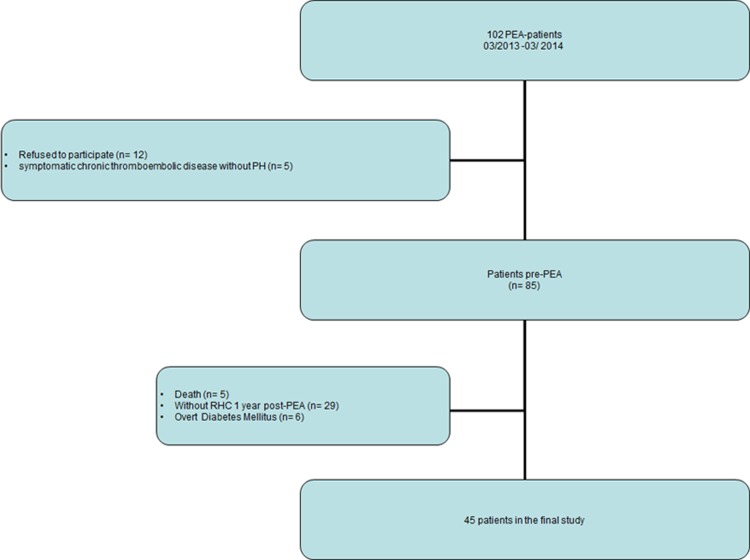
Flow chart of patient selection. RHC: right heart catheterization; PEA: pulmonary endarterectomy; PH: pulmonary hypertension.

All CTEPH patients undergoing PEA between March 2013 and March 2014 at the Department of Thoracic surgery, Kerckhoff-Clinic, Bad Nauheim, Germany were prospectively screened. After exclusion, 45 patients with complete baseline and one-year post-PEA hemodynamic data were analyze (Fig 1). Baseline and follow-up right heart catheter (RHC) were not mandatorily performed in-house, as the Kerckhoff-Clinic is a national referral center. Patients with symptomatic chronic thromboembolic disease and mean pulmonary artery pressure <25 mmHg at baseline were excluded [19] (Fig 1). Patients with a known history of diabetes mellitus or use of anti-diabetic treatment were excluded. All included patients gave written, informed consent, and the study was approved by the by the ethics committee of the Faculty of Medicine at the University of Giessen (Approval No. 31/13).

All CTEPH patients were diagnosed according to current guidelines at that time [20] and a multidisciplinary board including pulmonary physicians, PEA surgeons and pulmonary radiologists assessed operability. At inclusion, all patients had received oral anticoagulants for at least 3 months. All patients underwent PEA according to the protocol of the Kerckhoff-Clinic [21] and treatment with targeted PAH therapy was permitted without restrictions. Residual PH one year post PEA was defined by mPAP >25 mm Hg and PVR > 240 dyne*s/cm^5^ at rest [6] while the CTEPH type was classified by the surgical specimen as described previously [22].

### HbA1c testing

Venous blood samples were collected at the time of initial referral and were immediately analyzed in the laboratory of the Kerckhoff-Clinic.

HbA1c-levels were measured by Bio-Rad VARIANT™ II TURBO (Bio-Rad Laboratories, Inc. Hercules, California, USA) with a reference range of 29–42 mmol/mol. In addition, fasting plasma glucose, N-terminal fragment of pro-brain natriuretic peptide (NT-proBNP) and serum creatinine were measured. Glomerular filtration rate was calculated using the Chronic Kidney Disease-Epidemiology Collaboration (CKD-EPI) equation [23].

The investigator who determined HbA1c was unaware of the patient baseline parameters or clinical course and HbA1c-levels were not used to guide patient management or monitor the effects of treatment during the study period.

### Baseline parameters

Hemodynamic, 6-minute walk test (6MWT), echocardiography and cardio-pulmonary exercise testing (CPET) data from all included patients were collected before and after PEA. Right heart catheter (RHC) at baseline and follow-up were performed according current recommendations at that time [20]. However, as the Kerckhoff-Clinic is a national referral center, RHC were partly performed at referring centers. All patients performed a symptom-limited incremental CPET using a ramp protocol with an incremental rate of 5 to 15 W/min judged by the operator (Masterscreen®, Carefusion®) [24]. In all patients 6MWT was performed according to current guidelines and patients were instructed to walk at their own pace along a 30-m corridor while standard phrases were communicated [25]. Pulmonary artery systolic pressure (PASP) and tricuspid annular plane systolic excursion (TAPSE) were assessed by right heart echocardiography as described previously [26].

### Statistical analysis

Data are presented as mean ± standard deviation for normally distributed parameters. As appropriate the 2-tailed T-test, paired T-test, Mann-Whitney-U-test, Wilcoxon Signed Rank test or Pearson Chi-Square test was used to test for differences between groups. HbA1c was dichotomized at the cut-off indicative for the diagnosis of diabetes (47.5 mmol/mol) and of pre-diabetes (39 mmol/mol) as stated in the position statement of the American Diabetes Association (ADA) [27]. Linear regression analysis was used to assess associations of HbA1c with clinical parameters. All baseline parameters were analyzed in a univariate logistic regression model with residual PH as dependent variable. Then, backward stepwise multivariate logistic regression models were used to predict residual PH one-year post-PEA, considering all parameters of the univariate analysis with a p-value <0.15. Statistical analysis was performed using SPSS, version 21.0 (IBM, Armonk, NY).

## Results

### Baseline

45 CTEPH patients with a mean age of 62.7 ± 12.3 years and a mean body mass index of 27.3 ± 6.0 kg/m^2^ were included. Patients presented mostly in WHO functional class III with an impaired exercise capacity. At mean they had severe precapillary pulmonary hypertension with a mPAP of 43.6 ± 9.4 mmHg, PAWP of 9.9 ± 5.3 mmHg, PVR of 712.1 ± 520.4 dyne*s/cm^5^ and a substantially reduced CI. Right heart echocardiography showed an elevated PASP of 75.4 ± 24.1 mmHg and a reduced TAPSE of 17.6 ± 5.0 mm. At baseline the HbA1c was 39.8 ± 5.6 mmol/mol and NT-proBNP-levels were significantly elevated while GFR and serum creatinine were within the normal range. At time of inclusion 15 patients received monotherapy with pulmonary vasoactive therapies, mostly with phosphodiesterase type-5 inhibitors (Table 1).

**Table 1:** Baseline characteristics.

	Baseline	One-year post-PEA
Patients, n	45	
Male/Female	20/25	
Age (y)	62.7 ± 12.3	
BMI (kg/m^2^)	27.3 ± 6.0	
Laboratory		
HbA1c (mmol/mol)	39.8 ± 5.6	39.5 ± 4.4
GFR (l/min/m^2^)	81.9 ± 24.3	74.7 ± 25.1
Creatinine (mg/dl)	0.96 ± 0.3	0.99 ± 0.33
Fasting plasma glucose (mg/dl)	103.8 ± 30.4	102.1 ± 17.2
NT-proBNP (pg/ml)	966.0 [298.7–2286.3]	299.0 [124.0–474.7] ^#^
Hb (g/l)	14.5 ± 1.8	13.4 ± 1.8**
Hct (%)	42.9 ± 5.0	41.7 ± 4.6
WHO functional class, n (%)		‡t16
I	None	7 (15.6)
II	9 (20)	20 (44.4)
III	28 (62.2)	9 (20)
IV	8 (17.8)	None
RHC		
mPAP (mm Hg)	43.6 ± 9.4	28.5 ± 10.4*
RAP (mm Hg)	6.7 ± 3.4	6.5 ± 3.7
PVR (dyne*s/cm^5^)	712.1 ± 520.4	314.9 ± 233.7*
CI (l/min/m^2^)	2.4 ± 0.5	2.6 ± 0.6***
PAWP (mm Hg)	9.9 ± 5.3	10.8 ± 4.4
Echocardiography		
TAPSE (mm)	17.6 ± 5.0	17.1 ± 3.2
PASP (mm Hg)	75.4 ± 24.1	54.2 ± 22.4*
6MWD (m)	362.2 ± 133.9	434.7 ± 124.5*
VO_2_ peak (ml/min/kg)	12.4 ± 4.1	14.2 ± 3.9****
Co-morbidities, n (%)		
Dyslipidemia	5 (11.1)	Unchanged
Metabolic syndrome	5 (11.1)	Unchanged
Hypertension	23 (51.1)	Unchanged
Coronary heart disease	6 (13.3)	Unchanged
Renal insufficiency	8 (17.8)	Unchanged
Pulmonary vasoactive therapies, n (%)		
Phosphodiesterase type-5 inhibitors	11 (24.4)	1 (2.2)
Endothelin receptor antagonists	3 (6.7)	None
Prostanoids	1 (2.2)	None
Soluble guanylate cyclase stimulator	None	8 (17.8)
No pharmacological treatment	30 (66.7)	36 (80)
Jamieson-Type [22], n (%)	‡‡	
I	16 (37.2)	
II	10 (22.2)	
III	17 (37.8)	

Values represent mean ± SD or median [IQR]. CI: cardiac index, mPAP: mean pulmonary arterial pressure; PVR: pulmonary vascular resistance; RAP: right atrial pressure, PAWP: pulmonary arterial wedge pressure, TAPSE: tricuspid annular plane systolic excursion; PASP: pulmonary artery systolic pressure; 6MWD: six-minute walking distance; GFR: Glomerular filtration rate; HbA1c: Glycosylated hemoglobin A1c; VO_2_: oxygen uptake WHO: World Health Organization; NT-proBNP: N-terminal fragment of pro-brain natriuretic peptide; Hb: haemoglobin; Hct: haematocrit.

*p<0.001

**p = 0.003

***p = 0.04

****p = 0.04

^#^ p = 0.002 versus baseline. (* = paired T-test, ^#^ = Wilcoxon Signed Rank test)

‡n = 36

‡‡n = 43.

### One-year post pulmonary endarterectomy outcome

One-year post-PEA functional and hemodynamic parameters significantly improved and most patients were in WHO functional class II (44.4%). HbA1c, Glucose and serum creatinine remained unchanged while NT-proBNP and Hb were significantly reduced (Table 1). Residual PH was found in 24 patients with significantly elevated mPAP, PVR and NT-proBNP in comparison with non PH. In addition, a higher percentage of patients with residual PH presented in WHO functional class III and were treated notably with soluble guanylate cyclase stimulators (Table 2). No differences in HbA1c, Glucose or serum creatinine were evident in comparison of patients with residual and non-residual PH.

**Table 2:** Parameters one-year post-PEA according to residual PH.

	non PH	residual PH
Patients, n (%)	21 (46.7)	24 (53.3)
Laboratory		
HbA1c (mmol/mol)	37.6 ± 2.9	40.9 ± 4.8
GFR (l/min/m^2^)	81.1 ± 23.3	70.0 ± 25.8
Creatinine (mg/dl)	0.95 ± 0.28	1.03 ± 0.36
Fasting plasma glucose (mmol)	104.9 ± 22.4	100.1 ± 12.5
NT-proBNP (pg/ml)	217.0 [38–472.5] ^#^	389.9 [297.8–1077.6]
Hb (g/l)	13.9 ± 1.2	13.1 ± 2.1
Hct (%)	41.9 ± 2.7	41.6 ± 5.9
WHO functional class, n (%)	^‡^ ^†^	‡‡
I	5 (31.3)	2 (10)
II	11 (68.7)	9 (45)
III	None	9 (45)
IV	None	None
RHC		
mPAP (mm Hg)	20.3 ± 2.8*	35.7 ± 9.2
RAP (mm Hg)	5.2 ± 2.4**	7.6 ± 4.2
PVR (dyne*s/cm^5^)	170.5 ± 57.8*	435.3 ± 257.1
CI (l/min/m^2^)	2.8 ± 0.6	2.5 ± 0.6
PAWP (mm Hg)	9.2 ± 3.2**	12.2 ± 4.8
Echocardiography		
TAPSE (mm)	17.3 ± 3.0	16.9 ± 3.4
PASP (mm Hg)	43.0 ± 18.0	59.0 ± 22.9
6MWD (m)	478.8 ± 129.7	406.1 ± 115.4
VO_2_ peak (ml/min/kg)	15.5 ± 3.8	13.0 ± 3.7
Pulmonary vasoactive therapies, n (%)	None	9 (37.5)
Phosphodiesterase type-5 inhibitors	None	1 (4.2)
Soluble guanylate cyclase stimulator	None	8 (33.3)
Jamieson-Type [22], n (%)	Ұ	ҰҰ
I	6 (30)	10 (43.5)
II	4 (20)	6 (26.1)
III	10 (50)	7 (30.4)

Values represent mean ± SD or median [IQR]. For abbreviations see Table 1.

*p<0.001

**p = 0.02

***p = 0.023

****p = 0.004

^#^p = 0.026

^†^p = 0.006, versus residual PH (* = 2-tailed T-test, ^#^ = Man-Whitney-U-test, ^†^ = Pearson Chi-Square test)

‡n = 16

‡‡n = 20

Ұn = 20

ҰҰn = 23.

### Clinical relevance of HbA1c

Analyzing baseline characteristics were either dichotomized at HbA1c-level of 47.5 mmol/mol indicative of diabetes or 39 mmol/mol indicative of pre-diabetes [27]. Exercise capacity was more severely impaired in patients with HbA1c-levels > 47.5 mmol/mol displayed by a significantly lower peak VO_2_. Moreover, these patients had a slight but insignificant enhanced impairment of renal function. Interestingly, patients with HbA1c-levels > 39 mmol/mol and > 47.5 mmol/mol showed significant higher RAP in comparison (Table 3).

**Table 3:** Baseline Parameters according to HbA1c dichotomized at the cut-off indicative of diabetes or prediabetes according to the American Diabetes Association [27].

	HbA1c ≤ 47.5 mmol/mol	HbA1c > 47.5 mmol/mol	HbA1c ≤ 39 mmol/mol	HbA1c > 39 mmol/mol
Patients, n (%)	40 (89)	5 (11)	21 (47)	24 (53)
Laboratory				
HbA1c (mmol/mol)	38.4 ± 4.1*	51.0 ± 1.9	35.4 ± 2.7^†^	43.7 ± 4.5
GFR (l/min/m^2^)	83.4 ± 24.1	69.4 ± 25.5	88.9 ± 25.0	75.7 ± 22.5
Creatinine (mg/dl)	0.92 ± 0.29	1.1 ± 0.43	0.86 ± 0.23	1.02 ± 0.35
Fasting plasma glucose (mmol)	99.2 ± 24.4**	141.2 ± 48.8	95.0 ± 16.6	111.8 ± 37.2
NT-proBNP (pg/ml)	746.5 [284.0–1847.0]	3412 [1436.2–5021.5]	512.6 [175.0–1337.5]	1808 [425.2–3101.0]
Hb (g/l)	14.6 ± 1.7	14.1 ± 2.7	14.5 ± 1.9	14.5 ± 1.8
Hct (%)	43.3 ± 5.2	43.1 ± 6.4	42.9 ± 5.3	43.5 ± 5.2
WHO functional class, n (%)				
II	9 (22.5)	none	4 (19)	5 (21)
III	25 (62.5)	3 (60)	14 (66.7)	14 (58)
IV	6 (15)	2 (40)	3 (14.3)	5 (21)
RHC				
mPAP (mm Hg)	43.3 ± 9.0	45.6 ± 13.3	44.3 ± 8.7	42.9 ± 10.1
RAP (mm Hg)	6.4 ± 3.1***	11.0 ± 4.6	5.6 ± 2.2^†††^	7.9 ± 4.1
PVR (dyne*s/cm^5^)	698.9 ± 505.7	729.4 ± 441.6	582.8 ± 197.2	818.9 ± 668.3
CI (l/min/m^2^)	2.4 ± 0.5	2.0 ± 0.5	2.6 ± 0.5^††^	2.2 ± 0.5
PAWP (mm Hg)	9.7 ± 4.7	12.5 ± 10.3	9.5 ± 5.3	10.3 ± 5.5
Echocardiography				
TAPSE (mm)	17.6 ± 4.7	17.3 ± 8.5	18.7 ± 4.6	16.6 ± 5.3
PASP (mm Hg)	76.3 ± 22.9	68.3 ± 36.3	69.7 ± 23.3	79.8 ± 24.3
6MWD (m)	374.6 ± 133.6	265.0 ± 98.2	343.6 ± 127.0	377.6 ± 140.1
VO_2_ peak (ml/min/kg)	12.9 ± 4.0****	8.8 ± 2.8	13.2 ± 3.5	11.8 ± 4.5

Values represent mean ± SD or median [IQR]. For abbreviations see Table 1.

*p<0.001

**p = 0.003

***p = 0.02

****p = 0.035 versus HbA1c > 47.5 mmol/mol

^†^p<0.001

^††^p = 0.02

^†††^p = 0.03 versus HbA1c > 39 mmol/mol (* and ^†^ = 2-tailed T-test).

**Fig 2:**
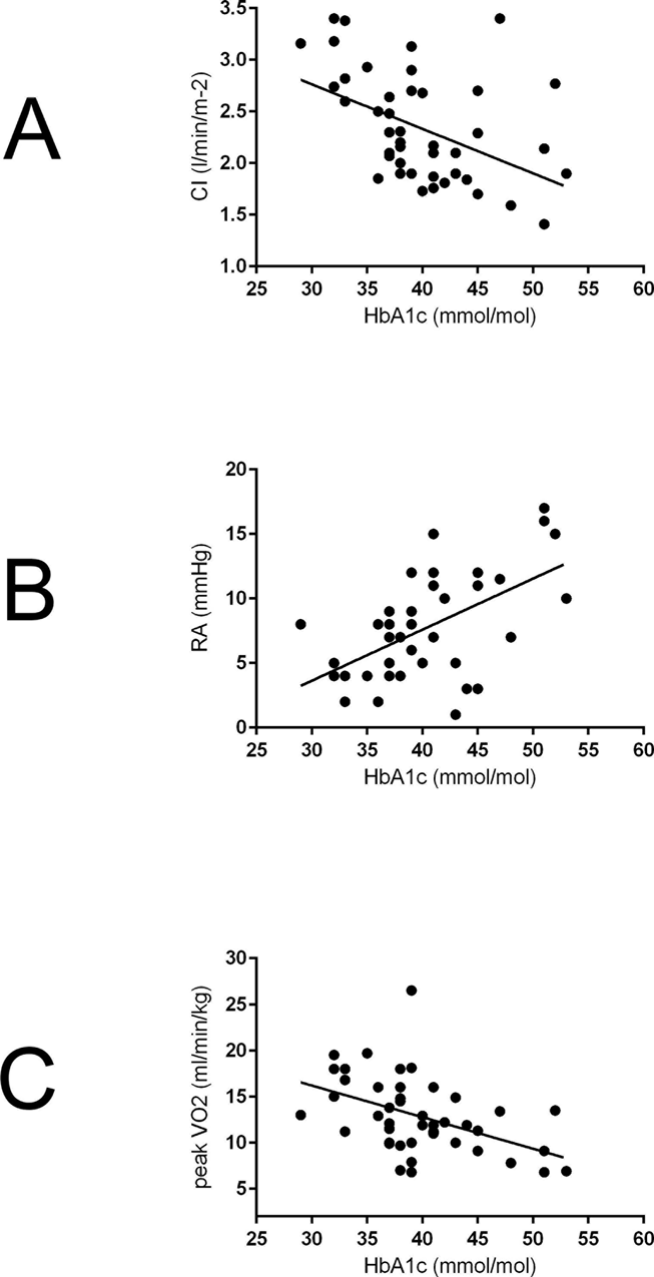
**Associations between baseline HbA1c and CI (r = 0.47, p = 0.001) (A), RAP (r = 0.37, p = 0.023) (B) and peak VO**_**2**_ **(r = 0.39, p = 0.015) (C).** For abbreviations see Table 1.

**Fig 3:**
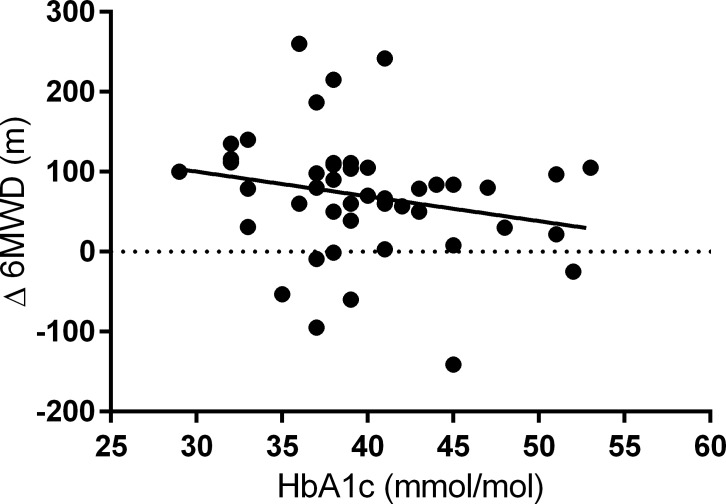
Association between baseline HbA1c-levels and the change of 6MWD (r = 0.32, p = 0.03). For abbreviations see Table 1.

Baseline HbA1c levels were correlated with baseline CI (r = 0.47, p = 0.001), RAP (r = 0.37, p = 0.023) and peak VO_2_ (r = 0.39, p = 0.015) (Fig 2). There were no significant associations between TAPSE (r = 0.28, p = 0.08), PASP (r = 0.11, p = 0.49), 6MWD (r = 0.10, p = 0.54) and other baseline parameters (S1 Table). In addition, baseline HbA1c-levels were significantly associated with the change of 6MWD (r = 0.32, p = 0.03; Fig 3) albeit not with the change of mPAP (r = 0.05, p = 0.75), PVR (r = 0.19, p = 0.23), CI (r = 0.28, p = 0.07) one-year post-PEA. Moreover, we analyzed patients without residual PH one-year post-PEA separately. In this cohort HbA1c was significantly associated with TAPSE one-year post-PEA (S2 Table).

Univariate logistic regression analysis revealed that baseline HbA1c-levels were not associated with the presence of residual PH one-year post-PEA (p = 0.22). Additional multivariate analysis including all parameters with p-value <0.15 of the univariate analysis and HbA1c (albeit not significant in the univariate model) revealed that HbA1c was not related to the hemodynamic outcome one-year post-PEA with a HR of 0.91 (95% CI for HR 0.76–1.01, p = 0.31). The multivariate analysis identified baseline peak VO_2_ as a significant predictor of residual PH with a HR of 0.78 (95% CI for HR 0.62–0.99, p = 0.04), while other parameters failed to serve as independent predictors of the functional outcome one-year post-PEA in the stepwise backward multivariable model (Table 4).

**Table 4:** HbA1c levels and baseline parameters as predictors of residual PH one-year post-PEA.

	Univariate model		Multivariate model^#^	
	HR (95% CI)	p-value	HR (95% CI)	p-value
HbA1c (mmol/mol)	1.07 [0.96–1.20]	0.22	-	-
mPAP (mm Hg)	1.06 [0.99–1.14]	0.11	-	-
RAP (mm Hg)	1.01 [0.84–1.23]	0.89	-	-
PVR (dyne*s/cm^5^)	1.00 [0.99–1.01]	0.92	-	-
CI (l/min/m^2^)	0.50 [0.16–1.62]	0.25	-	-
PAWP (mm Hg)	1.03 [0.92–1.16]	0.63	-	-
TAPSE (mm)	0.89 [0.77–1.02]	0.09	-	-
PASP (mm Hg)	1.02 [0.99–1.05]	0.17	-	-
VO_2_ peak (ml/min/kg)	0.78 [0.62–0.98]	0.03	0.78 [0.62–0.99]	0.04
6MWD (m)	0.99 [0.99–1.02]	0.25	-	-
Nt-pro BNP (pg/ml)	1.00 [1.00–1.01]	0.43	-	-
WHO functional class			-	-
II	Reference		-	-
III	2.31 [0.48–11.12]	0.30	-	-
IV	6.00 [0.72–49.84]	0.10	-	-
Jamieson-Type [22]	0.65 [0.32–1.3]	0.22	-	-
Hb (g/l)	1.06 [0.76–1.46]	0.74	-	-
Hct (%)	1.03 [0.92–1.16]	0.60	-	-
Fasting plasma glucose (mmol)	1.03 [1.0–1.07]	0.07	-	-

HR: hazard ratio; CI: confidence interval; For abbreviations see Table 1.

^#^: backward stepwise logistic regression including variables with a p-value <0.15 in the univariate model.

## Discussion

The present study prospectively evaluated HbA1c as a biomarker in a selected cohort of operable CTEPH patients and investigated its association with the outcome one-year post-PEA. An association of HbA1c with baseline hemodynamic parameters and exercise capacity was found. However, baseline HbA1c levels did not predict the hemodynamic outcome one-year post-PEA.

Our study included operable CTEPH patients with severely impaired pulmonary hemodynamics and low exercise capacity mostly in WHO functional class III. Hemodynamic parameters and exercise capacity of our cohort mirrored the typical findings of patients with operable CTEPH prior to PEA with a substantial impairment of right ventricular function as well as elevated PVR, mPAP and reduced CI [28–32]. One-year post-PEA hemodynamic parameters, exercise capacity and Nt-proBNP improved significantly, albeit 25 patients (49%) of our cohort exhibited a residual PH. In line with previously published data a significant improvement of the functional outcome one-year post-PEA was observed [3, 4]. However, as described in our study, the individual outcome after PEA differed with 10% to 24% of patients remaining symptomatic [4, 33] and up to 35% were presenting with residual PH [6, 30, 34]. The established diagnosis of diabetes mellitus type II was present in 6 patients, which were excluded from the analysis. Nevertheless, the low prevalence of established diagnosis of diabetes type II in our cohort is in accordance with international registry data reporting diabetes type II in approximately 5.3% of CTEPH patients [35]. However, using the ADA criteria 5 additional patients who did not have the diagnosis of diabetes mellitus yet, presented with indicative high HbA1c-levels > 47.5mmol/mol.

Interestingly, our data indicated for the first time that HbA1c-levels prior to PEA correlated with baseline RAP, CI and peak VO_2_ in operable CTEPH patients, although similar hemodynamic parameters (apart from RAP) and RV systolic function were found in comparison of low HbA1c-levels (< 47.5mmol/mol) with elevated HbA1c-levels. In addition, patients with HbA1c levels > 39 mmol/mol [27], presented with a further impaired CI and higher RAP in comparison. The significant association of HbA1c with baseline RAP in CTEPH underlines the presence of diastolic dysfunction due to stiffer right ventricles in a more profound glycemic environment, as described for PAH patients previously [36]. In addition, impaired glucose metabolism is potentially influencing CI in CTEPH by aggravating impaired myocardial relaxation and increased myocardial stiffness as seen in diabetic cardiomyopathy [37] or metabolic disease in PAH [38]. Similar to our finding of an association of glucose intolerance in operable CTEPH with worse exercise capacity as assessed by peak VO_2_, Pugh and coworkers demonstrated a trend to a correlation of baseline HbA1c-levels with functional capacity (assessed by 6MWD) in PAH [13]. Moreover, our study showed a weak but significant association of HbA1c-levels with the change of 6MWD one-year post-PEA. However, up to now, no robust associations of the altered glucose metabolism and 6MWD have been reported in PH patients. Zamanian and coworkers described no significant difference of the 6MWD between PAH patients with and without IR [14]. Further, our data suggest that HbA1c is more elevated in advanced disease, although differences in WHO functional class, mPAP and PVR were not statistically significant.

Despite these significant associations to baseline parameters, HbA1c was not significantly correlated to outcome parameters post-PEA in univariate and multivariate analysis. One can speculate that the influence of the glycemic environment pre-PEA on secondary small-vessel arteriopathy and RV dysfunction plays only a minor role one-year post-PEA and/or is not reflected by HbA1c directly. In addition, our cohort presented with predominantly proximal disease as reflected by the absence of Jamieson-Type IV patients. Furthermore, the relatively high number of residual PH in our study potentially results from incomplete removal of obstructions and/or a varying degree of reverse right ventricular remodeling after surgery rather than from concomitant small vessel disease. Thus, the prognostic impact of impaired glucose metabolism as a solitary marker is comparably moderate. Other pre-operative predictors of post-operative outcome such as mPAP [7], serum creatinine level, the number of involved segments, PVR and gender [39] are useful complementary parameters to the currently reported HbA1c-levels.

A limitation of our study is the lack of systematic evaluation post-PEA to differentiate between non-removable material or secondary microvascular disease as major cause of residual PH. In addition, register analysis featured an enhanced influence of diabetes as a risk factor in IPAH patients rather than operable CTEPH [35]. Therefore, in case of inoperable CTEPH with distal disease and pronounced secondary small-vessel arteriopathy the association of impaired glucose metabolism assessed by HbA1c with pulmonary hemodynamics and long-term outcome remains unknown and in need of further investigations. Pathophysiologically, the obstructive remodeling of pulmonary arteries in CTEPH involves inflammation, in situ thrombosis and endothelial dysfunction [40]. Inflammatory mediators play a key role in the pathological mechanisms of CTEPH partly mediating the obstructive remodeling of pulmonary arteries [40] and being associated with RV dysfunction [41]. Yet, cytokines have not been linked to impaired glucose metabolism to date. However, in patients with PAH and idiopathic PAH, albeit not in CTEPH, various studies have extensively analyzed the clinical relevance of HbA1c and an association of impaired glucose metabolism with disease severity was found [11, 13, 36]. Moreover, IR has been identified as an important risk factor for disease progression and is associated with a poorer survival in PAH [14]. IR is linked to pro-inflammatory cytokines with significant contribution to the pathophysiology of PAH [14] and is associated with the development of pulmonary vascular disease in mice [42]. Furthermore, animal models have shown that impaired glucose metabolism and IR have a direct impact on the pulmonary vasculature and the pulmonary smooth muscle cell (SMC) leading to higher RV pressures, enhanced RV hypertrophy and peripheral pulmonary arterial muscularization [43]. In addition, the glycemic environment induces endothelial dysfunction with an impaired relaxant response through dysregulations in the NO pathway in diabetic rats [44]. We did not asses IR as our study was not designed to evaluate these parameter, therefore future trails addressing this issue are warranted.

Limitations of the study are the moderate sample size, the lack of prospective inclusion of inoperable CTEPH patients and the overall low prevalence of impaired glucose metabolism in our cohort. As we are a national referral center, the one-year follow-up visit was not mandatorily performed in our center and accounts for the dropout rate of 29 patients. In addition, the rate of residual PH was higher than reported in the literature. The relatively high rate of residual PH is not fully understood. The rate is influenced by many factors, including the type of pre-surgical CTEPH (proximal vs. distal), the severity of pre-surgical PH (possible reflection of micro-vascular disease) and the different underlying confounders that lead to CTEPH (e.g. simple recurrent deep vein thrombosis and pulmonary embolism vs. splenectomy vs. infected pacemaker leads etc.). We also believe that the selection criteria for the current observation might result in an enrichment of patients who are prone to have a comparably high proportion of residual PH after surgery. Lastly, a selection bias might have played a role, in that patients with residual PH (i.e. symptomatic patients) are more likely to be followed up by means of RHC.

## Conclusions

In conclusion, this is the first study to describe an association of HbA1c-levels with pulmonary hemodynamic parameters and exercise capacity in a selected cohort of operable CTEPH patients. Our preliminary results indicate that in these patients impaired glucose metabolism as assessed by HbA1c is of clinical significance. However, HbA1c did not predict hemodynamic outcome one-year post-PEA in our cohort.

## Supporting Information

S1 Table Correlations of HbA1c with baseline parameter using linear regression analysis. (DOCX)

S2 Table Correlations of HbA1c with hemodynamic and functional outcome in patients with non-PH one-year post-PEA using linear regression analysis. (DOCX)
