# Supplementary material for: The Clinical Significance of HbA1c in Operable Chronic Thromboembolic Pulmonary Hypertension
Source: PLoS One. 2016 Mar 31;11(3):e0152580. doi: 10.1371/journal.pone.0152580 (PMC4816563; doi:10.1371/journal.pone.0152580)
Supplement: S1 Table — (DOCX) [file pone.0152580.s001.docx]

**S1 Table. Correlations between HbA1c with baseline parameter using linear regression analysis.**

|  | Baseline parameters | |  | | | | | | | | | | | | | | | | | | |
| --- | --- | --- | --- | --- | --- | --- | --- | --- | --- | --- | --- | --- | --- | --- | --- | --- | --- | --- | --- | --- | --- |
|  | Hemoglobin (g/l) | | Hematocrit (%) | | GFR (l/min/m2) | | | Creatinine (mg/dl) | | | Fasting plasma glucose (mmol) | | NT-proBNP (pg/ml) | | | mPAP (mm Hg) | | | RAP (mm Hg) | | |
|  | r | p | r | p | r | | p | r | p | | r | p | r | p | | r | | p | r | p | |
| HbA1c (mmol/mol) | 0.01 | 0.99 | 0.07 | 0.66 | 0.21 | | 0.16 | 0.27 | 0.08 | | 0.49 | 0.001 | 0.35 | 0.14 | | 0.01 | | 0.99 | 0.37 | 0.023 | |
|  | Baseline parameters | |  | |  | | |  | | |  | |  | | |  | | |  | | |
|  | PVR (dyne*s/cm5) | | CI (l/min/m2) | | PAWP (mm Hg) | | | TAPSE (mm) | | | PASP (mm Hg) | | 6MWD (m) | | | VO_2_ peak (ml/min/kg) | | |  | | |
|  | r | p | r | p | r | p | | r | | p | r | p | r | | p | r | p | |  |  |  |
| HbA1c (mmol/mol) | 0.22 | 0.16 | 0.47 | 0.001 | 0.14 | 0.38 | | 0.28 | | 0.08 | 0.11 | 0.49 | 0.10 | | 0.54 | 0.39 | 0.015 | |  |  |  |

For abbreviations see Table 1.
