# Supplementary material for: The Clinical Significance of HbA1c in Operable Chronic Thromboembolic Pulmonary Hypertension
Source: PLoS One. 2016 Mar 31;11(3):e0152580. doi: 10.1371/journal.pone.0152580 (PMC4816563; doi:10.1371/journal.pone.0152580)
Supplement: S2 Table — (DOCX) [file pone.0152580.s002.docx]

**S2 Table. Correlations of HbA1c with hemodynamic and functional outcome in patients with non-PH one-year post-PEA using linear regression analysis**

For abbreviations see Table 1.

|  | Baseline parameters | |  | | | | | | | | | | | | | | | | | | |
| --- | --- | --- | --- | --- | --- | --- | --- | --- | --- | --- | --- | --- | --- | --- | --- | --- | --- | --- | --- | --- | --- |
|  | Hemoglobin (g/l) | | Hematocrit (%) | | GFR (l/min/m2) | | | Creatinine (mg/dl) | | | Fasting plasma glucose (mmol) | | NT-proBNP (pg/ml) | | | mPAP (mm Hg) | | | RAP (mm Hg) | | |
|  | r | p | r | p | r | | p | r | p | | r | p | r | p | | r | | p | r | p | |
| HbA1c (mmol/mol) | 0.36 | 0.11 | 0.39 | 0.08 | 0.22 | | 0.34 | 0.30 | 0.18 | | 0.37 | 0.10 | 0.28 | 0.22 | | 0.26 | | 0.26 | 0.23 | 0.38 | |
|  | Baseline parameters | |  | |  | | |  | | |  | |  | | |  | | |  | | |
|  | PVR (dyne*s/cm5) | | CI (l/min/m2) | | PAWP (mm Hg) | | | TAPSE (mm) | | | PASP (mm Hg) | | 6MWD (m) | | | VO_2_ peak (ml/min/kg) | | |  | | |
|  | r | p | r | p | r | p | | r | | p | r | p | r | | p | r | p | |  |  |  |
| HbA1c (mmol/mol) | 0.15 | 0.54 | 0.46 | 0.001 | 0.09 | 0.71 | | 0.53 | | 0.02 | 0.08 | 0.77 | 0.03 | | 0.91 | 0.29 | 0.23 | |  |  |  |
